# Trends in cancer imaging by indication, care setting, and hospital type during the COVID‐19 pandemic and recovery at four hospitals in Massachusetts

**DOI:** 10.1002/cam4.4183

**Published:** 2021-08-06

**Authors:** Ottavia Zattra, Anthony Fraga, Nancy Lu, Michael S. Gee, Raymond W. Liu, Michael H. Lev, James A. Brink, Sanjay Saini, Min Lang, Marc D. Succi

**Affiliations:** ^1^ Harvard Medical School Boston Massachusetts USA; ^2^ Department of Radiology Massachusetts General Hospital Boston Massachusetts USA; ^3^ Medically Engineered Solutions in Healthcare Incubator Innovation in Operations Research Center (MESH IO) Massachusetts General Hospital Boston Massachusetts USA

**Keywords:** cancer, COVID‐19 pandemic, imaging, medical, neoplasms, tomography, X‐ray computed

## Abstract

**Background:**

We aimed to investigate the effects of COVID‐19 on computed tomography (CT) imaging of cancer.

**Methods:**

Cancer‐related CTs performed at one academic hospital and three affiliated community hospitals in Massachusetts were retrospectively analyzed. Three periods of 2020 were considered as follows: pre‐COVID‐19 (1/5/20–3/14/20), COVID‐19 peak (3/15/20–5/2/20), and post‐COVID‐19 peak (5/3/20–11/14/20). 15 March 2020 was the day a state of emergency was declared in MA; 3 May 2020 was the day our hospitals resumed to non‐urgent imaging. The volumes were assessed by (1) Imaging indication: cancer screening, initial workup, active cancer, and surveillance; (2) Care setting: outpatient and inpatient, ED; (3) Hospital type: quaternary academic center (QAC), university‐affiliated community hospital (UACH), and sole community hospitals (SCHs).

**Results:**

During the COVID‐19 peak, a significant drop in CT volumes was observed (−42.2%, *p *< 0.0001), with cancer screening, initial workup, active cancer, and cancer surveillance declining by 81.7%, 54.8%, 30.7%, and 44.7%, respectively (*p *< 0.0001). In the post‐COVID‐19 peak period, cancer screening and initial workup CTs did not recover (−11.7%, *p *= 0.037; −20.0%, *p *= 0.031), especially in the outpatient setting. CT volumes for active cancer recovered, but inconsistently across hospital types: the QAC experienced a 9.4% decline (*p* = 0.022) and the UACH a 41.5% increase (*p* < 0.001). Outpatient CTs recovered after the COVID‐19 peak, but with a shift in utilization away from the QAC (−8.7%, *p* = 0.020) toward the UACH (+13.3%, *p* = 0.013). Inpatient and ED‐based oncologic CTs increased post‐peak (+20.0%, *p* = 0.004 and +33.2%, *p* = 0.009, respectively).

**Conclusions:**

Cancer imaging was severely impacted during the COVID‐19 pandemic. CTs for cancer screening and initial workup did not recover to pre‐COVID‐19 levels well into 2020, a finding that suggests more patients with advanced cancers may present in the future. A redistribution of imaging utilization away from the QAC and outpatient settings, toward the community hospitals and inpatient setting/ED was observed.

## Graphical abstract

Between March and November 2020, the use of CTs for cancer screening and initial cancer workup was significantly lower than in pre‐COVID‐19 times. Also, during this time, more cancer‐related imaging was performed at community hospitals, in the ED and in the inpatient setting, than at the large academic center and outpatient. The findings are concerning for inappropriate healthcare resource utilization, and the chance that more patients with advanced cancer may present post‐pandemic.

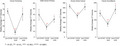

## 1 INTRODUCTION

To manage the COVID‐19 pandemic, medical institutions have shifted their focus toward critically ill COVID‐19 patients, minimizing non‐essential services to curb transmissions.^1^ Declines in available resources across different specialties have made accessing medical care difficult for non‐COVID‐19 patients.^2^

Oncologic care has been particularly affected by COVID‐19. At the beginning of the pandemic, individuals with cancer were identified to be at high risk for contracting COVID‐19 and experiencing severe disease, owing in part to their immunocompromised status.^3^, ^4^ In response to this finding, cancer centers globally began to reduce their care delivery, transitioning to telemedicine appointments, delaying procedures, and modifying treatment and surveillance schedules.^5^, ^6^ Many providers grappled with the challenge of delivering routine treatment while reducing virus exposure to patients and themselves.^7^ In other instances, patients chose to avoid in‐person treatments and visits, for fear of contracting the virus at the doctor's office.^8^ Furthermore, cancer center operations were affected by supply chain interruptions, staff reassignments, and fewer available facilities owing to the conversion of hospital spaces to COVID‐19 units.^5^

Altered standards of cancer care due to the pandemic have been far‐reaching: a study performed across 20 large American healthcare institutions revealed a significant drop in all cancer‐related patient encounters in April 2020.^9^ Another study found outpatient visit volumes in spring 2020 to be lower than 2019 volumes by 60%–70%, and it also uncovered a decline in mastectomies, colectomies, and prostatectomies.^10^ International studies registered lower numbers of chemotherapy appointments, radiation therapy sessions, and cancer‐related admissions in spring 2020.^11^, ^12^, ^13^, ^14^ Finally, cancer screening was severely affected by the pandemic: lung cancer screening decreased by more than 70% between March and May 2020,^15^ cervical cancer screenings by 78%,^16^ colorectal cancer screenings by 85%,^9^ and mammograms by up to 92%.^9^, ^17^, ^18^

### 1.1 Objectives

Few studies have explored the effects of the pandemic on cancer imaging after the lockdown of March–May 2020. In this study, we examine cancer imaging utilization at one academic hospital and three affiliated community hospitals in Massachusetts during both the March–May peak and a post‐peak period extending through November 2020. We specifically analyze computed tomography (CT) imaging, which is the dominant imaging modality for cancer care. We also analyze the differential effects of modified healthcare operations on distinct stages of cancer care: screening, surveillance, initial diagnostic work‐up, and active cancer. Finally, we examine variations in imaging utilization based on care setting (outpatient vs. inpatient vs. ED), as well as hospital type (academic vs. university‐affiliated community vs. rural). We hypothesize that imaging‐based screening, diagnosis, and management of cancer‐related disease were severely impacted by the COVID‐19 pandemic, with effects lasting beyond the initial spring lockdown, and that these effects may be unevenly distributed across stages of cancer care, care settings, and hospital types.

## 2 METHODS

### 2.1 Design

The study use of aggregate data was compliant with the Health Insurance Portability and Accountability Act (HIPAA) and was approved with exemption by our Institutional Review Board (IRB). We conducted a retrospective time series analysis of all CT scans performed at one single, large academic institution and three affiliated community medical centers in Massachusetts between 5 January 2020 and 14 November 2020. Our main academic hospital is a 1017‐bed, urban quaternary academic center (QAC), seeing approximately 50,000 inpatients, 100,000 ED patients, and 1.5 million outpatients per year. Among the three affiliated community centers, one is a 273‐bed suburban university‐affiliated community hospital (UACH); the remaining two meet federal criteria for sole community hospitals (SCH) and offer 25 and 19 beds each to two separate island communities off the southern coast of the state. Data from the two SCHs were pooled into one hospital setting category for the analysis.

### 2.2 Setting

**FIGURE 1:**
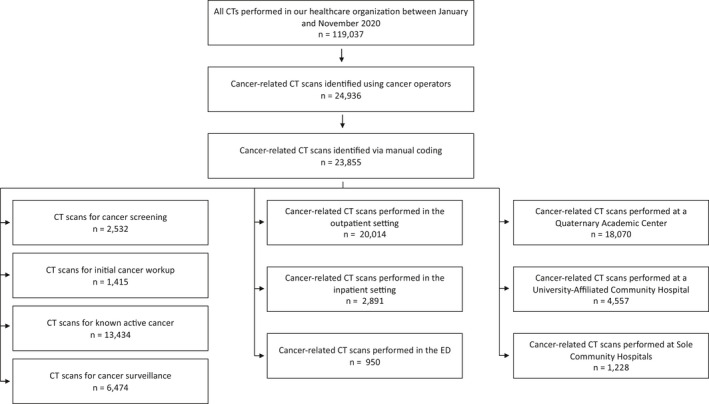
Data analysis algorithm

Data regarding each individual CT scan performed across the four hospitals were extracted from the electronic medical record (Epic Systems), yielding 119,037 entries during the 11‐month study period. Cancer‐related CT scans were first identified by a structured search of pre‐defined and standardized institutional cancer operators in the order requisition field of each exam (“malignant,” “neoplasm,” “metastasis,” “mets,” “metastatic,” “surveillance,” “staging,” “malignancy,” “cancer,” “mass,” “lump,” “tumor,” “tumour”). CTs were subsequently manually screened to ensure only oncology‐related imaging was considered. During the manual screening, CTs were also coded into four imaging indication categories depending on the stage of cancer care: cancer screening (e.g., annual low‐dose lung cancer CTs, familial syndromes, etc.); initial work‐up of suspected cancer or cancer rule‐out (e.g., all suspected first time cancers, workup of new unknown masses/lumps/abnormal labs, suspected recurrences of patients with a remote treated history of cancer); known active cancer (e.g., staging/restaging and imaging for any reason in patients with active cancer, generally undergoing treatment); and scheduled surveillance (e.g., no active cancer and not receiving treatment, but with a history of previously treated cancer). Data were also classified according to hospital setting (QAC, UACH, and SCHs) and care setting (inpatient, outpatient, and emergency department [ED]) (Figure 1).

Details regarding statewide public health information and legislation were obtained from State websites and used to designate three distinct time periods for the study: pre‐COVID‐19 (5 January 2020–14 March 2020), COVID‐19 peak (15 March 2020–2 May 2020), and post‐COVID‐19 peak (3 May 2020–14 November 2020). 10 March was the date a state of emergency in Massachusetts was declared; because our analysis was conducted on a weekly basis and 10 March 2020 happened to be a weekday, the week of 10 March was included in the pre‐COVID‐19 period. 3 May was the date our institution resumed non‐urgent imaging after the initial lockdown.

### 2.3 Statistical methods

For our analysis, we first calculated the number of cancer‐related CTs performed each week. Weeks were defined as Sunday–Saturday. We then calculated means and standard deviations of weekly imaging volumes for each of the three study periods. Weeks 1–11 were considered to represent the pre‐COVID‐19 period (5 January 2020–14 March 2020), Weeks 11–17 were considered to represent the COVID‐19 peak (15 March 2020–2 May 2020), Weeks 18–45 were considered to represent the post‐COVID‐19 peak (3 May 2020–14 November 2020). Finally, the pre‐COVID‐19 period was set as the pre‐pandemic baseline volume of cancer‐related CTs. Each mean weekly volume for the COVID‐19 peak and post‐COVID‐19 peak periods was compared to the baseline via percentage decrease computation and student's *t* test. The analysis was performed across imaging indications, care settings, and hospital types. Statistical analysis was performed using GraphPad Prism (GraphPad) and Excel (Microsoft).

## 3 RESULTS

### 3.1 Trends in cancer‐related CT volumes during the COVID‐19 peak

**FIGURE 2:**
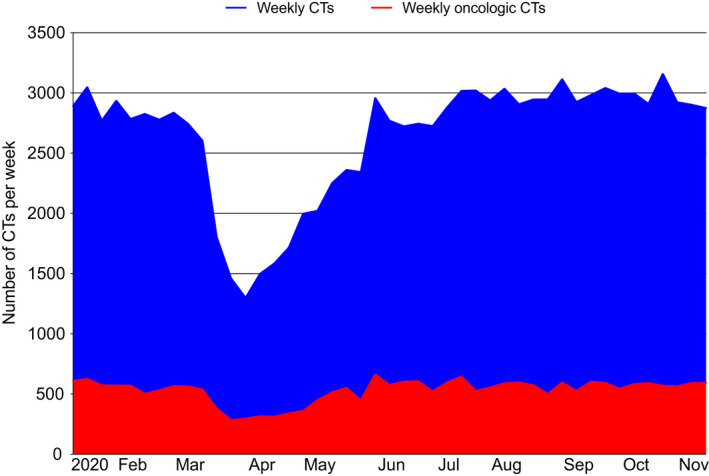
Weekly volumes of all computed tomography (CTs) and oncologic CTs from 5 January 2020 to 14 November 2020

After a declaration of emergency was announced in Massachusetts on 10 March 2020, cancer‐related weekly CT volumes declined drastically from a pre‐COVID‐19 weekly average of 566 CTs to 328 average weekly CTs during the COVID‐19 peak. This equaled a 42.2% drop (*p *< 0.0001; Table 1; Figure 2). The downtrend in cancer‐related CT volumes paralleled that of all CT volumes at our institution (−42.5%, *p* < 0.0001). The rate of decline of cancer‐related CTs matched that of all CTs, and thus the proportion of CTs that was cancer‐related remained constant (pre‐COVID‐19 vs. COVID‐19 peak: 20.1% vs. 20.3%).

**TABLE 1:** Cancer‐related computed tomography (CT) volumes as compared to all CT volumes and by imaging indication, hospital type, and care setting during three periods of 2020

	Pre‐COVID‐19 peak (baseline studies per week) (Mean ± SD)	COVID−19 peak (studies per week) (Mean ± SD)	Percentage of baseline (%)	*p* value^a^	Post‐COVID‐19 peak (studies per week) (Mean ± SD)	Percentage of baseline (%)	*p* value^a^
All CT volume							
All CT	2823.00 ± 119.92	1623.71 ± 234.08	57.5%	<0.0001	2837.18 ± 272.36	100.5%	0.825
Oncologic CT	566.40 ± 36.07	327.57 ± 34.69	57.8%	<0.0001	567.79 ± 49.47	100.2%	0.926
By imaging indication							
Cancer screening	70.30 ± 6.86	12.86 ± 6.23	18.3%	<0.0001	62.11 ± 16.34	88.3%	0.037
Initial work‐up	39.80 ± 9.62	18.00 ± 3.92	45.2%	<0.0001	31.82 ± 5.61	80.0%	0.031
Active cancer	316.80 ± 19.48	219.57 ± 21.01	69.3%	<0.0001	311.75 ± 30.73	98.4%	0.556
Surveillance	139.50 ± 16.81	77.14 ± 13.68	55.3%	<0.0001	162.11 ± 24.80	116.2%	0.004
By care setting							
Outpatient	487.70 ± 38.51	278.43 ± 30.97	57.1%	<0.0001	471.00 ± 43.92	96.6%	0.272
Inpatient	61.00 ± 9.76	33.00 ± 5.60	54.1%	<0.0001	73.21 ± 11.32	120.0%	0.004
ED	17.70 ± 5.29	16.14 ± 3.89	91.2%	0.495	23.57 ± 5.43	133.2%	0.009
By hospital setting							
QAC^b^	441.70 ± 36.48	262.29 ± 29.16	59.4%	<0.0001	422.04 ± 32.64	95.5%	0.153
UACH^c^	98.70 ± 9.08	51.43 ± 10.23	52.1%	<0.0001	114.64 ± 21.49	116.2%	0.003
SCHs^d^	26.00 ± 8.16	13.86 ± 6.20	53.3%	0.003	31.11 ± 6.78	119.6%	0.098

^a^ Compared to pre‐COVID‐19 peak period.

^b^ Quaternary academic center.

^c^ University‐affiliated community hospital.

^d^ Sole community hospitals.

**FIGURE 3:**
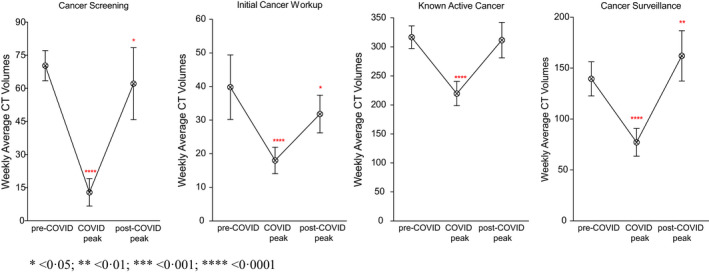
Weekly Average computed tomography volumes by imaging indication across the three study periods

Weekly CT volumes for all stages of cancer care experienced a significant reduction during the COVID‐19 peak: cancer screening CTs decreased by 81.7% (*p *< 0.0001), initial workup CTs by 54.8% (*p *< 0.0001), active cancer CTs by 30.7% (*p *< 0.0001), and surveillance scans by 44.7% (*p* < 0.0001, Figure 3) as compared to the baseline. Across care settings, weekly CT volumes performed in the outpatient and inpatient settings decreased significantly during the COVID‐19 peak. Cancer‐related CTs performed in the ED during the peak did not decline (−8.8%, *p* = 0.495). Across hospital types, the SCHs were the only locations that preserved stable CT volumes across imaging indications during the COVID‐19 peak, and that only saw significant declines in cancer screening (−76.4%, *p *< 0.0001, Table S1).

### 3.2 Trends in cancer‐related CT volumes in the post‐peak period

After the first wave of the pandemic peaked in April 2020, our healthcare system resumed normal imaging operations on 3 May, and reinstituted non‐emergent deferred care 2 weeks later.^19^ With these policy changes, the overall volume of cancer‐related CTs performed across our healthcare organization recovered to pre‐COVID‐19 levels during the post‐peak period (+0.2%, *p *= 0.926, Table 1). However, the recovery was inconsistent across imaging indications, care settings, and hospital types.

Computed tomography volumes for cancer screening and for initial workup did not recover to pre‐COVID‐19 levels in the post‐peak period (−11.7% from baseline, *p* = 0.037; −20% from baseline, *p *= 0.031, Figure 3). The outpatient setting was particularly affected and experienced declines from baseline of 14% for cancer screening CTs (*p *= 0.024) and 43.2% for initial workup CTs (*p *< 0.001). CT volumes for active cancer recovered to baseline levels in the post‐peak period (−1.6%, *p *= 0.556). However, the recovery was not uniform across hospital types: in the post‐peak period, the QAC experienced a loss in weekly CTs for active cancer by almost 10% (*p* = 0.022), whereas the UACH saw a significant increase in this imaging category by 41.5% (*p* < 0.001). Across care settings, CT volumes for active cancer recovered to pre‐COVID‐19 levels in the outpatient and inpatient settings, and they significantly increased by 58.1% in the ED (*p* < 0.001). CT volumes for scheduled surveillance in the post‐peak period not only recovered but rebounded by +16.2% as compared to baseline (*p* = 0.004). This volume growth was observed across all three hospital types (+11.2% at the QAC, *p* = 0.026; +32.1% at the UACH, *p* = 0.031; +71.4% at the SCHs, *p *= 0.003) and in the outpatient setting (+15.6%, *p *= 0.012).

In the outpatient setting, cancer‐related CT volumes in the post‐peak period recovered to baseline (−3.4%, *p *= 0.272). However, the recovery was inconsistent across hospital types: the QAC saw a decline in outpatient CTs by 8.7% from baseline (*p *= 0.020, Table S1), while the UACH saw a growth of the same by 13.3% (*p *= 0.013). In the inpatient setting, CTs experienced a 20% increase in the post‐peak period (*p *= 0.004): this increase occurred specifically at the QAC (+14.5% from baseline, *p *= 0.040) and at the UACH (+80.8% from baseline, *p *< 0.001). In the ED, cancer‐related CTs saw a 33.2% increase from baseline in the post‐peak period (*p *= 0.009), with volumes at the QAC ED in particular experiencing a 34.7% increase (*p *= 0.014).

## 4 DISCUSSION

Our study adds to the literature suggesting that COVID‐19 severely impacted cancer imaging care in 2020.^5^, ^6^ We found significant declines in cancer CT screening and cancer diagnosis that persistent beyond the initial wave of the pandemic. In addition, we discovered that cancer‐related CT volumes decreased in outpatient clinics and quaternary medical centers and increased in EDs, inpatient settings, and community hospitals.

During the COVID‐19 peak, CT volumes for all oncological imaging indications decreased significantly, with cancer screening and diagnostic workup volumes plummeting by more than 50%. After the peak, our institution experienced a recovery of cancer‐related imaging, albeit inconsistently across stages of cancer care. While imaging for active cancer and cancer surveillance recovered to pre‐COVID‐19 levels, cancer screening and diagnostic workup volumes did not fully recover (down 11.7% and 20% from baseline, respectively). Multiple factors may explain the incomplete recovery. From the delivery side, social distancing measures resulted in dramatic reductions in care capacity, with imaging appointments often limited to patients with active cancer. From the patient side, behavioral shifts have led to patients avoiding hospitals for fear of contracting COVID‐19 or because of financial stress. Many individuals opted to delay check‐ups and to handle symptoms at home.^8^ Outpatient care has been particularly impacted, which is reflected in our data. Our findings suggest a contraction in healthcare delivery for new malignancies, potentially resulting in a surge of new and more advanced cancer diagnoses in the upcoming months to years.

Our study also revealed a shift in CT utilization from outpatient clinics to EDs and inpatient units: in the post‐peak period, CTs performed in the ED and in the inpatient setting rose by 33% and 20%, respectively. Of concern, the growth in ED‐based CTs was mostly driven by patients with active cancer, whose rate of imaging utilization in our EDs increased by 58% in the post‐peak period. These trends underscore the severe impact of even a few months of interrupted care, with higher acuity patients presenting for care. Furthermore, the ED never experienced the initial drop in imaging volumes during the COVID‐19 peak, and thus saw high utilization by cancer patients throughout the pandemic. While the ED remains a dependable source of cancer care for many, this healthcare setting is often not optimized to tend to the complex medical needs of oncology patients. Cancer‐related ED visits, in fact, are often driven by preventable symptoms, frequently lead to inpatient stays, and tend to cost more than visits for other chronic medical conditions.^20^ Furthermore, ED‐based oncology care delivery can often be inefficient, as demonstrated by a study revealing longer turnaround times for non‐acute cancer CTs than CTs performed for other causes.^21^

Finally, our study uncovered a redistribution of imaging utilization away from the QAC toward the community hospitals. This trend was mostly driven by increases in inpatient‐based imaging and imaging for active cancers at the UACH, which grew by 81% and 42% from baseline, respectively. The redistribution correlated with hospital capacity, as the two SCHs experienced the least amount of disruption to their cancer care operations. A possible explanation for this observation was that urban hospitals with greater service capability directed more resources toward COVID‐19 care, and thus greatly reduced subspecialty oncology care. Thus, patients living in metropolitan areas would preferentially present at, or be directed to, the suburban community hospital as opposed to the large university center downtown. On the other hand, patients living in remote rural communities had no choice but to continue presenting to their local hospital for care, thus rural community centers likely had to maintain operational volumes close to pre‐pandemic levels. In any case, the shift in imaging use away from cities and large hospitals is symptomatic of inappropriate resource utilization across healthcare systems, as resource‐limited medical centers may often be unsuited to meet the needs of complex oncology patients.

To the best of our knowledge, this is the first study analyzing the differential impact of COVID‐19 on cancer CT imaging by imaging indications, care setting, and hospital type. Our results are concordant with prior clinical literature: cancer care delivery across all stages of medical management experienced a setback in 2020. A decline in screenings across malignancy types was reported by a number of studies: in the United States, lung cancer screening CTs experienced a drop,^15^, ^22^ and so did screening mammograms,^18^ and colonoscopies.^23^ Another US‐based study recorded decreases in five different modalities of cancer screening (low dose CT, pap test, colonoscopy, prostate‐specific antigen, and mammography) during the COVID‐19 peak, with subsequent declines in ensuing diagnoses.^24^ The management of active cancers also experienced a worrisome drop, with both US‐based and international studies finding high rates of cancellations and delays for chemotherapy, radiation therapy, surgeries, and cancer‐related admissions.^10^, ^11^, ^13^, ^14^ One study recorded a rise in palliative care enrollment during 2020, which suggests that support for pain and symptom management was available to cancer patients, but also that more individuals might have opted out of anticancer therapies in favor of comfort measures.^11^ Importantly, none of these studies conducted investigations past the summer of 2020, and thus lacked the ability to record the longer term effects of the pandemic on cancer care. The present study remedies this by analyzing cancer imaging during the pandemic until the end of 2020.

Considering the disquieting effects of the pandemic on cancer care, many predict significant rises in cancer rates and deaths on the horizon.^25^, ^26^ The consequences of reducing oncology care have already begun to be captured. One study recorded higher numbers of lung nodules suspicious for cancer (Lung‐RADS 4) in the screening CTs performed after the COVID‐19 peak, underscoring the effects of just a few months’ delay in care.^22^ A UK‐based analysis estimated increases in death rates by 4%–16% as a result of diagnostic delays, corresponding to more than 3000 excess deaths in 5 years.^27^ Finally, a study found a significant association between delays in treatment initiation and 5‐year mortality for the four most common types of cancer in the USA.^28^ The drop in cancer care delivery highlighted by our study will likely have similar implications for cancer patients in our network. Quantifying the effect of care delays will be important for better systemwide resource allocation and planning. It will also be important to assess the detrimental effects of oncology care reductions on the mental well‐being of patients and providers. Surveys conducted during 2020 have revealed high numbers of physicians experiencing anxiety and depression, with treatment delays being a major driver of concern.^29^, ^30^ Similarly, cancer patients have expressed widespread concern regarding delays and changes in care.^8^ In the upcoming months and years, as the pandemic hopefully slows, it is paramount that we support the mental health of all those involved in cancer care, in order to fully reverse the negative impact of COVID‐19 and to strive once more for the best possible cancer outcomes.

Our study has a few limitations. First, our analysis focused exclusively on CT imaging. This choice was dictated by their widespread use in oncology, and by their relative volume stability during 2020: CTs were one of the least affected imaging modalities during the pandemic,^17^, ^31^ therefore significant changes in their volumes were more likely to reflect real variations in healthcare utilization. However, analyzing other imaging modalities might provide a more nuanced understanding of the impact of COVID‐19 on oncology. For example, at our institution, mammogram screening is often patient‐scheduled, thus may reveal different trends than provider‐ordered CTs. Second, changes in CT volumes were calculated in relation to the pre‐COVID‐19 CT volumes of January–March 2020. It is possible that these baseline volumes might not be representative of imaging volumes from prior years. Third, our institution is in a state that experienced many COVID‐19 cases from the very beginning of the pandemic, and therefore our results might not be as generalizable to less‐affected regions. Lastly, our analysis was only conducted across four hospitals, of which only one was an academic medical center. Our sample size was, therefore, small and this may limit the generalizability of our findings.

In conclusion, the COVID‐19 pandemic has had a severe impact on the delivery of oncology care in the United States. Our study substantiated a general decline in cancer care during the COVID‐19 peak, followed by an inconsistent recovery of care in the post‐peak period favoring established patients with active cancers and disadvantaging cancer screenings and initial diagnoses. A redistribution of CT utilization away from outpatient and academic center‐based imaging toward inpatient/ED and community hospital‐based imaging was also observed, which is concerning for optimizing resource allocation and appropriate care. Future studies should aim at quantifying the longer term effects of the pandemic by extending the analysis period into 2021 and beyond. Furthermore, other measures of cancer care utilization should be explored, such as billing for chemotherapy and radiation therapy. These investigations will help healthcare systems across our country to prepare for potential surges in cancer‐related imaging and diagnoses.

## ETHICS STATEMENT

Approved with exemption by the Institutional Review Board.

## CONFLICT OF INTEREST

The authors declare no relevant conflict of interest.

## AUTHOR CONTRIBUTION

Ottavia Zattra, BSPH: Data curation, formal analysis, methodology, and writing—original draft. Anthony Fraga, BS: Data curation and writing—review & editing. Nancy Lu, BS: Data curation and writing—review & editing. Michael S. Gee, MD PhD: Methodology, supervision, validation, and writing—review & editing. Raymond W. Liu, MD: Methodology, supervision, validation, and writing—review & editing. Michael H. Lev, MD: Methodology, supervision, validation, and writing—review & editing. James A. Brink, MD: Methodology, supervision, validation, and writing—review & editing. Sanjay Saini, MD: Methodology, supervision, validation, and writing—review & editing. Min Lang, MD MSc: Formal analysis, methodology, and writing—review & editing. Marc D. Succi MD: Conceptualization, data curation, formal analysis, methodology, supervision, and writing––review & editing.

## Supporting information

Table S1

## Data Availability

The data that support the findings of this study are available from the corresponding author upon reasonable request.
