# Supplementary material for: Trends in cancer imaging by indication, care setting, and hospital type during the COVID‐19 pandemic and recovery at four hospitals in Massachusetts
Source: Cancer Med. 2021 Aug 6;10(18):6327–35. doi: 10.1002/cam4.4183 (PMC8420511; doi:10.1002/cam4.4183)
Supplement: Supplementary file 1 — Table S1 [file CAM4-10-6327-s001.docx]

Supplement Table 1: Oncologic CT volumes by Hospital Type and Imaging Indication, Hospital Type and Care Setting, and Care Setting and Imaging Indication during three different periods of 2020.

|  | Pre-COVID-Peak  (studies per week)  *(Mean ± SD)* | COVID-Peak  (studies per week)  (*Mean ± SD)* | Percentage of Baseline  (%) | P-value^a^ | Post-COVID-Peak (studies per week)  *(Mean ± SD)* | Percentage of Baseline  (%) | P-value^b^ |
| --- | --- | --- | --- | --- | --- | --- | --- |
| By Imaging Indication & Hospital Type |  |  |  |  |  |  |  |
| CANCER SCREENING |  |  |  |  |  |  |  |
| Quaternary Academic | 38·60 ± 3·44 | 7·57 ± 3·91 | 19·6% | <0·0001 | 34·93 ± 10·11 | 90·5% | 0·104 |
| UACH^b^ | 22·60 ± 4·17 | 4·00 ± 1·73 | 17·7% | <0·0001 | 19·04 ± 7·32 | 84·2% | 0·073 |
| SCH^c^ | 9·10 ± 3·07 | 2·14 ± 2·12 | 23·6% | <0·0001 | 8·11 ± 3·68 | 89·1% | 0·416 |
| INITIAL WORK-UP |  |  |  |  |  |  |  |
| Quaternary Academic | 24·50 ± 6·80 | 11·71 ± 3·40 | 47·8% | <0·001 | 20·79 ± 4·79 | 84·8% | 0·138 |
| UACH^b^ | 13·40 ± 4·77 | 4·43 ± 1·51 | 33·1% | <0·001 | 8·96 ± 3·21 | 66·9% | 0·018 |
| SCH^c^ | 1·90 ± 1·29 | 1·86 ± 1·35 | 97·7% | 0·949 | 2·04 ± 1·48 | 107·1% | 0·786 |
| ACTIVE CANCER |  |  |  |  |  |  |  |
| Quaternary Academic | 265·50 ± 27·08 | 180·29 ± 20·65 | 67·9% | <0·0001 | 240·54 ± 25·04 | 90·6% | 0·022 |
| UACH^b^ | 40·40 ± 10·66 | 32·57 ± 6·92 | 80·6% | 0·086 | 57·18 ± 12·09 | 141·5% | <0·001 |
| SCH^c^ | 10·90 ± 5·47 | 6·71 ± 2·81 | 61·6% | 0·058 | 14·04 ± 4·86 | 128·8% | 0·132 |
| SURVEILLANCE |  |  |  |  |  |  |  |
| Quaternary Academic | 113·10 ± 12·06 | 62·71 ± 11·46 | 55·4% | <0·0001 | 125·79 ± 20·09 | 111·2% | 0·026 |
| UACH^b^ | 22·30 ± 8·08 | 11·29 ± 5·68 | 50·6% | 0·005 | 29·46 ± 8·67 | 132·1% | 0·031 |
| SCH^c^ | 4·00 ± 2·16 | 3·14 ± 2·67 | 78·6% | 0·497 | 6·86 ± 2·56 | 171·4% | 0·003 |
| By Imaging Indication & Care Setting |  |  |  |  |  |  |  |
| CANCER SCREENING |  |  |  |  |  |  |  |
| Outpatient | 64·80 ± 7·07 | 9·43 ± 4·96 | 14·5% | <0·0001 | 55·75 ± 16·52 | 86·0% | 0·024 |
| Inpatient | 4·50 ± 2·37 | 3·00 ± 2·45 | 66·7% | 0·230 | 5·18 ± 2·16 | 115·1% | 0·439 |
| ED | 1·10 ± 0·99 | 0·43 ± 0·79 | 39·0% | 0·142 | 1·18 ± 1·16 | 107·1% | 0·840 |
| INITIAL WORK-UP |  |  |  |  |  |  |  |
| Outpatient | 24·70 ± 6·85 | 8·14 ± 2·91 | 33·0% | <0·0001 | 14·04 ± 3·12 | 56·8% | <0·001 |
| Inpatient | 7·20 ± 3·49 | 5·14 ± 1·07 | 71·4% | 0·108 | 8·86 ± 3·01 | 123·0% | 0·203 |
| ED | 7·90 ± 3·60 | 4·71 ± 1·11 | 59·7% | 0·024 | 8·93 ± 2·89 | 113·0% | 0·430 |
| ACTIVE CANCER |  |  |  |  |  |  |  |
| Outpatient | 275·40 ± 19·30 | 193·71 ± 18·01 | 70·3% | <0·0001 | 259·29 ± 29·87 | 94·2% | 0·064 |
| Inpatient | 34·60 ± 11·54 | 17·43 ± 5·06 | 50·4% | 0·001 | 41·71 ± 9·58 | 120·6% | 0·103 |
| ED | 6·80 ± 2·49 | 8·43 ± 3·21 | 124·0% | 0·284 | 10·75 ± 3·05 | 158·1% | <0·001 |
| SURVEILLANCE |  |  |  |  |  |  |  |
| Outpatient | 122·80 ± 17·23 | 67·14 ± 11·81 | 54·7% | <0·0001 | 141·93 ± 22·96 | 115·6% | 0·012 |
| Inpatient | 14·70 ± 5·25 | 7·43 ± 3·10 | 50·5% | 0·003 | 17·46 ± 4·04 | 118·8% | 0·154 |
| ED | 2·00 ± 1·25 | 2·57 ± 1·27 | 128·6% | 0·375 | 2·68 ± 1·94 | 133·9% | 0·220 |
| By Care Setting & Hospital Type |  |  |  |  |  |  |  |
| OUTPATIENT |  |  |  |  |  |  |  |
| Quaternary Academic | 373·00 ± 34·97 | 222·29 ± 25·34 | 59·6% | <0·0001 | 340·68 ± 29·14 | 91·3% | 0·020 |
| UACH | 89·30 ± 8·37 | 42·86 ± 8·71 | 48·0% | <0·0001 | 101·21 ± 19·58 | 113·3% | 0·013 |
| SCH | 25·40 ± 7·96 | 13·29 ± 6·16 | 52·3% | 0·003 | 29·11 ± 6·57 | 114·6% | 0·207 |
| INPATIENT |  |  |  |  |  |  |  |
| Quaternary Academic | 56·00 ± 9·70 | 28·71 ± 4·07 | 51·3% | <0·0001 | 64·14 ± 10·52 | 114·5% | 0·040 |
| UACH | 4·90 ± 1·97 | 4·29 ± 3·68 | 87·5% | 0·698 | 8·86 ± 3·56 | 180·8% | 0·0002 |
| SCH | 0·10 ± 0·32 | 0·00 ± 0·00 | NA^d^ | 0·343 | 0·11 ± 0·31 | 107·1% | 0·952 |
| ED |  |  |  |  |  |  |  |
| Quaternary Academic | 12·70 ± 4·22 | 11·29 ± 2·56 | 88·9% | 0·404 | 17·11 ± 5·00 | 134·7% | 0·014 |
| UACH | 4·40 ± 2·07 | 4·29 ± 1·70 | 97·4% | 0·903 | 4·57 ± 2·57 | 103·9% | 0·835 |
| SCH | 0·50 ± 0·97 | 0·57 ± 0·53 | 114·3% | 0·849 | 1·89 ± 1·57 | 378·6% | 0·003 |

^a^Compared to pre-COVID-peak period

^b^University-Affiliated Community Hospital

^c^Sole Community Hospital

^d^Not Applicable
